# The elevated metabolic cost of walking at preferred speeds of healthy elderly on treadmills compared to overground is not related to increased self-reported anxiety

**DOI:** 10.1007/s00421-023-05138-y

**Published:** 2023-01-20

**Authors:** Sauvik Das Gupta, Herre Faber, Dinant Kistemaker, Maarten Bobbert

**Affiliations:** 1 Department of Human Movement Sciences, Faculty of Behavioural and Movement Sciences, Amsterdam Movement Sciences, Vrije Universiteit Amsterdam, Amsterdam, The Netherlands; 2 Human Movement Biomechanics Research Group, Department of Movement Sciences, Biomedical Sciences Group, KU Leuven, Leuven, Belgium; 3 Faculty of Health, Nutrition and Sports, The Hague University for Professional Education, The Hague, The Netherlands

**Keywords:** Healthy aging, Gross cost of walking, Net cost of walking, Anxiety, High and low treadmills

## Abstract

**Purpose:**

To investigate whether the elevation in metabolic cost of walking on treadmills compared to overground for healthy elderly is related to self-reported anxiety and if changes in self-reported anxiety are related to changes in heart rate.

**Methods:**

We measured overground preferred walking speed, oxygen consumption rate and heart rates during rest and walking, and self-reported anxiety in 10 elderly (mean age 69.5 ± 3.1 years, 8 males and 2 females). At their preferred speed, the participants first walked overground, then on a high treadmill, and then on a low treadmill. Gross and Net metabolic costs of walking were calculated from the rates of oxygen consumption.

**Results:**

Gross and net metabolic cost of walking were higher (*p* < 0.05) on high treadmill (net cost: 2.64 J kg^−1^ m^−1^) and low treadmill (net cost: 2.68 J kg^−1^ m^−1^) compared to overground (net cost: 2.44 J kg^−1^ m^−1^), and the same was true for heart rate. There were no significant differences (*p* > 0.05) in metabolic costs and heart rates between the two treadmill conditions. Self-reported anxiety was higher on the high treadmill compared to overground (*p* = 0.004) and compared to low treadmill (*p* = 0.02). We found no significant difference (*p* > 0.05) for self-reported anxiety between overground and the low treadmill.

**Conclusion:**

These results show that treadmill walking cannot be adequately generalized to overground walking. The differences found in metabolic cost on treadmills compared to overground were not related to differences in self-reported anxiety. Furthermore, the changes in heart rate are not related to changes in self-reported anxiety.

## Introduction

Metabolic energy expenditure during walking is an important factor that determines whether a person is able to satisfactorily perform activities of daily life. It has been previously shown in the literature that Metabolic Cost of Walking (MCoW), defined as the metabolic energy expended per kilogram of body mass per meter travelled, is about 15% higher in elderly compared to young (Das Gupta et al. 2019). When MCoW is expressed as Gross Cost of Walking (GCoW) the elevation is in the order of ~ 0.3 J kg^−1^ m^−1^ (*d* = 0.65), and when MCoW is expressed in terms of Net Cost of Walking (NCoW), the elevation is in the order of ~ 0.4 J kg^−1^ m^−1^ (*d* = 1.00) (Das Gupta et al. 2019). Most of the studies on MCoW have been conducted on treadmills (e.g., Gaesser et al. 2018; Malatesta et al. 2004, 2003; Mian et al. 2006; Peterson and Martin 2010). Previously, we compared MCoW in healthy young and elderly during both overground and treadmill walking (Das Gupta et al. 2021). We found no effects of age per se on MCoW during overground walking. However, NCoW during treadmill walking was increased by 0.6 J kg^−1^ m^−1^ compared to overground walking in elderly; in young adults, there was no elevation in NCoW on the treadmill compared to overground walking. Previously, Parvataneni et al. (2009) measured MCoW in elderly and showed that there was an excess metabolic energy consumption of 23% on a treadmill compared to overground, accompanied by a 6% increase in heart rate on the treadmill compared to overground. This suggests that the elevated MCoW of elderly compared to young reported in the literature may be caused by elderly using a different gait on treadmill compared to overground. For example, elderly could walk on a treadmill with more co-contraction of hip, knee, and ankle muscles (Tudor-Locke et al. 2021) to increase stability. Obviously, co-contraction causes increased MCoW, as shown previously (Peterson and Martin 2010; Hortobágyi et al. 2011; Ortega and Farley 2015; Mian et al. 2006; Tudor-Locke et al. 2021; Piche et al. 2021). This then prompts the question what the trigger is for elderly to use a different gait on treadmill compared to overground.

It has been proposed that anxiety could be a trigger for elderly to walk differently on treadmill than overground (Parvataneni et al. 2009), especially if elderly are unfamiliar with treadmill walking. The anxiety, in turn, could be caused by the fact that visual flow is unnatural on a treadmill (Dal et al. 2010; Martin and Li 2017; Murray et al. 1985); after all on a treadmill, there is a discrepancy between visual information and vestibular and other proprioceptive information. On the other hand, anxiety could also be caused by the treadmill’s height above the floor level. In the literature, it has been observed in elderly that walking heart rate relative to rest is higher on treadmill than overground (Greig et al. 1993; Parvataneni et al. 2009) and the suggestion that anxiety is increased on a treadmill in elderly is based on this observation. However, rather than reflecting increased anxiety, increased heart rate might be directly related to increased MCoW. After all, it is known that in healthy individuals, metabolic rate and cardiac output are closely correlated (e.g., Guyton et al. 1973). Therefore, the theory proposed in the literature that increased MCoW on a treadmill is due to changes in gait pattern, and/or increased muscle co-contraction, which are triggered by anxiety, may not be correct. It could also be that elderly have higher MCoW on a treadmill due to gait changes arising from (unknown) biomechanical or/and neuromuscular issues that are not related to increased anxiety. This would elevate their heart rate simply because higher MCoW requires higher cardiac output.

The aim of the present study was to determine whether increased MCoW of elderly on a treadmill compared to overground is related to self-reported anxiety. For this aim, we first set out to replicate our result from the previous study (Das Gupta et al. 2021), i.e., that MCoW of elderly was increased on our treadmill compared to overground. Additionally, we asked our participants to indicate their anxiety level on a Visual Analogue Scale (VAS) and examined whether the increased MCoW on our elevated treadmill compared to overground was accompanied by increased self-reported anxiety. Second, we studied whether self-reported anxiety was reduced when our elderly walked on a floor-level treadmill compared to our elevated treadmill and was in the same range as that of overground. Furthermore, we checked whether MCoW on this floor-level treadmill differed, compared to the MCoW on the elevated treadmill and to overground. We did so, because in our previous study of MCoW in elderly and young adults on treadmill and overground, we used a treadmill that was elevated by 60 cm (Das Gupta et al. 2021), while other authors of comparable studies generally used a treadmill at floor level. It could be that in our previous study, the extra height of the treadmill was a cause for increased anxiety and thereby triggered gait changes resulting in increased MCoW in our elderly. During all experiments, we also measured heart rates and examined whether heart rates were related to self-reported anxiety.

## Methods

### Characteristics of participants and ethics statement

We recruited 10 healthy elderly (mean age 69.5 ± 3.1 years, 8 males and 2 females) and measured their anthropometrics and overground preferred walking speed (PWS). All the participants were physically active and carried out their normal day-to-day activities without any assistance. None of the participants were actively involved in any kind of special strength or endurance training. They had at least once walked previously on a treadmill in their life, but never on a high (from ground level) treadmill. We excluded participants if they had any pathological condition (like chronic heart disease, diabetes, past surgeries or prosthesis in the lower limbs and feet, neuromuscular disabilities), were on medication (including sleeping aids or antidepressants which may impact mobility) or had experienced a fall (we used the general definition of a fall: losing one’s balance and collapsing to the ground) in the past 6 months. We excluded all these conditions or factors as they might have an influence on the MCoW. Written informed consent was obtained from each participant. Before the start of the experimental protocol, body mass and height were measured. Lower limb lengths from the greater trochanter to the malleolus and from the greater trochanter to the foot were also measured to obtain the leg lengths. The ethical review committee of the Faculty of Behavioural and Movement Sciences of the Vrije Universiteit Amsterdam approved the experimental protocol.

### Protocol

#### Pre-experimental factors

**Fig. 1:**
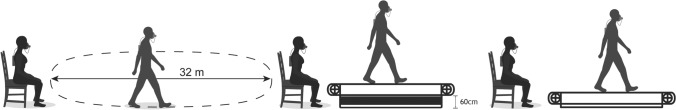
Schematic overview of the energy expenditure measurements during the experimental protocol. Resting Metabolic Rate (RMR) was measured, while the participants were seated for 5 min on a chair preceding each of the overground and the treadmill walking trials. During the overground trial, participants walked continuously along the schematically drawn oval track with 32 m straights interconnected by two half-circles of 4 m radius. During the treadmill trials, the participants first walked on the high treadmill (placed at a height of 60 cm from the ground) and then on a normal floor-level treadmill at their imposed overground preferred walking speeds

To ensure that diet did not have an effect on MCoW prior to the experiments, participants were told to have only a light breakfast (i.e., moderate intake of carbohydrates and fats, minimalize intake of protein and fibre-rich foods as much as possible; food intake was listed). They were also asked to not consume any caffeine or alcohol-containing products and to refrain from smoking tobacco until the experiments were finished. We checked and ensured that the participants adhered to both these requests from us. The participants walked overground along an oval track with 32 m straights, interconnected by two half-circles of 4 m radius inside a laboratory (see Fig. 1). After walking for 5 min, participants completed three full laps. To establish PWS participants were asked to walk at the speed, they would normally walk while going from their home to a supermarket. PWS was calculated as the average walking speed during the last three laps. Subsequently, PWS was imposed on a treadmill. Two treadmills were used for the study: one treadmill (from ForceLink BV, Culemborg, The Netherlands) placed at a height of 60 cm above the ground (henceforth referred to as high treadmill) and one standard treadmill (from Motek Medical BV, Houten, The Netherlands) with the walking surface at 15 cm above the ground (henceforth referred to as low treadmill). Handrails were not provided to allow the participants to have a natural arm-swing. The participants were secured to an overhead rail with a chest-harness while walking on the treadmills. To give adequate rest to the participants, we ensured that there were at least 20 min between two consecutive trials. We had a fixed order of our walking trials overground and on the treadmills, which we will discuss in the following section.

#### Measurement of self-reported anxiety, heart rate, and metabolic cost of walking

To test the idea that anxiety is leading to a higher MCoW while walking on a treadmill, we measured both heart rate as well as self-reported anxiety using a VAS. We measured heart rate using a Polar heart rate chest sensor (from Polar, Kempele, Finland) to test the suggestion done in the literature that the increased heart rate when walking on a treadmill indicates an increased anxiety, which is then suggested to lead to an increase in MCoW. However, heart rate is a main determinant of the cardiac output and as such is also directly related to physical exertion. Since the main goal of this study was to investigate if found differences in MCoW (indicating differences in physical exertion) could be explained by anxiety, we also used an anxiety measurement that is not (e.g., like sweat production and body temperature) confounded by physical exertion: the “anxiety thermometer” (Houtman and Bakker 1989) which is basically a VAS. This anxiety thermometer has been extensively validated to be a measure of a person’s state and trait anxiety (Almazrouei et al. 2022; Kantor et al. 2001; Malian et al. 2021; Thibodeau et al. 2012; Williams et al. 2010; Bourne and Vladeanu 2011; Elwood et al. 2012; Ertuğ et al. 2017; Hulsman et al. 2010; Arora et al. 2010) and has been used in several studies to measure self-reported anxiety (Englert and Seiler 2020; Harris et al. 2022; Linssen et al. 2022; Payne et al. 2019) including in studies that involved various locomotor tasks (Cañal-Bruland et al. 2010; Nibbeling et al. 2012; Nieuwenhuys and Oudejans 2010; Pijpers et al. 2003, 2005, 2006).

Oxygen consumption (${\dot{\text{V}}\text{O}}_{2}$) and carbon dioxide production rates (${\dot{\text{V}}\text{CO}}_{2}$) were measured using a portable Cosmed K4b2 (from Cosmed, Rome, Italy) breath-by-breath indirect calorimetry device and a face-mask. The Cosmed K4b2 was always calibrated according to the manufacturer’s guidelines before collecting any data. The participants sat in a chair for 5 min in a relaxed state, and the resting oxygen consumption (Resting Metabolic Rate, RMR) and resting heart rate level were measured (see Fig. 1). For reasons provided elsewhere (Weyand et al. 2009), we chose to measure their resting values in a seated posture (see also: Das Gupta et al. 2021). The RMR measured immediately before a walking trial was used to calculate the NCoW for the corresponding walking trial. We chose to include the GCoW measurements alongside the standard NCoW measurements to account for any variations in the baseline metabolic rate that might occur during the walking protocols (see Courter et al. 2022). GCoW (in J kg^−1^ m^−1^) was calculated from the rate of oxygen consumption using Lusk’s equation (Lusk 1923) and PWS as follows:

1 $${\text{GCoW}} = (15962 + 5155 \cdot {\text{RER}}) \cdot {\dot{\text{V}}\text{O}}_{2} \cdot {\text{PWS}}^{ - 1} ,$$

where $(15962 + 5155 \cdot {\text{RER}})$ is the energetic equivalent of oxygen (in J l^−1^), RER is the Respiratory Exchange Ratio (dimensionless), ${\dot{{V}}\text{O}}_{2}$ is the oxygen consumption rate (in l kg^−1^ s^−1^), and PWS is the average walking speed during the last three overground laps (in m·s^−1^). NCoW (in J kg^−1^ m^−1^) was obtained by inserting net ${\dot{\text{V}}\text{O}}_{2}$, i.e., the difference in ${\dot{\text{V}}\text{O}}_{2}$ between walking and sitting. The gross heart rate values were reported from the Polar heart rate monitor directly in beats per minute (bpm).

#### Preparation

Before any walking exercise, we explained the anxiety thermometer to our elderly participants. We explained them that ‘0’ on the scale means no anxiety and a ‘10’ on the scale means extreme anxiety. Then, we told our participants to mark a ‘X’ with a pen on the thermometer corresponding to how anxious they felt after they completed the overground or the treadmill walking tasks. All the instructions were given in the participants native language (i.e., Dutch). Then, the particpants were fitted with the portable Cosmed device and a face-mask, and with the heart rate sensor.

#### Experimental protocol

After fitting of the equipment, the participants first completed the overground walking protocol and then walked for 15 min on the high treadmill and then for another 15 min on the low treadmill at their overground PWS, while we measured their oxygen consumption and heart rate levels (see Fig. 1). We kept the walking protocol identical to our previous study, because we wanted to replicate our own results of MCoW overground and on a treadmill (Das Gupta et al. 2021). Just like in that study, on both the treadmills, the speed was gradually increased over the first 2 min until it matched the participants’ overground PWS and then kept constant. At the end of the trial (i.e., after 15 min of walking), the speed was slowly reduced to zero, and then, the measurement of oxygen consumption and heart rates were stopped. Before each of the walking trials, we repeated the (seated) resting oxygen consumption and heart rate measurements.

From the overground oxygen consumption rate and heart rate data, the last 30 s of data were always discarded, and then, the remaining last 2 complete minutes of data were used to calculate MCoW and heart rate levels. A similar duration of oxygen consumption and heart rate data from the treadmills, exactly corresponding to the minutes of the overground trials, were used to calculate the MCoW and heart rate levels on the treadmills. Immediately after each of the walking sessions, the participants filled in their anxiety level on the anxiety thermometer (0–10 with 0.1 point increment) (Houtman and Bakker 1989).

### Statistical tests

For GCoW, NCoW, heart rate, and self-reported anxiety level, statistical comparisons were performed between the three walking conditions (overground, high treadmill, and low treadmill). Since the purpose of the study was to explain differences in MCoW between treadmill and overground walking with differences in self-reported anxiety between treadmill and overground walking, we chose to look at intra-individual (within-subjects) differences in MCoW, heart rates, and self-reported anxiety levels. As all the comparisons were within-subjects, the assumption for the equality of variances was always met. Furthermore, we also averaged the time histories of gross metabolic power over participants and graphically displayed means and Standard Error of the Mean (SEM) as a function of time for overground, high treadmill, and low treadmill walking conditions before doing a full-blown statistical analysis. We then conducted a non-parametric Friedman RM-ANOVA test, and in case of statistically significant results (*p* < 0.05), post hoc Durbin–Conover pairwise comparisons were conducted to check which two walking conditions differed significantly from one another. The open-source software JAMOVI (version 1.8.2.0) was used for all the statistical tests and the default value of α = 0.05 was chosen as the level of statistical significance.

## Results

### Age and anthropometric measurements

In Table 1, we have listed the mean values and standard deviations (mean ± SD) of age, anthropometrics, and overground PWS (on average 1.4 ± 0.1 m·s^−1^) that was imposed on the treadmills. Furthermore, none of our older adults encountered a fall during the course of the experiments.

**Table 1:** Age, anthropometric parameters, and PW*S* of healthy elderly

	Healthy elderly
Age (years)	69.5 ± 3.1
Body mass (kg)	75.6 ± 14.2
Height (m)	1.72 ± 0.10
Lower limb length up to malleolus (m)	0.83 ± 0.07
Lower limb length up to foot (m)	0.90 ± 0.07
Overground PWS (m·s^−1^)	1.4 ± 0.1

### Metabolic cost of walking, heart rate, and anxiety levels

**Fig. 2:**
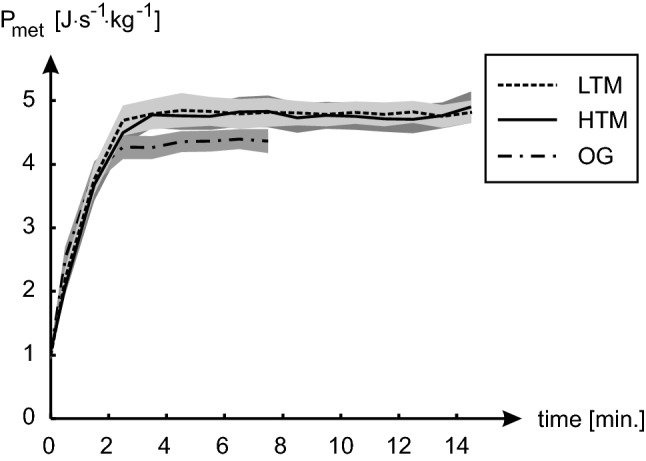
Overview of gross metabolic power (*P*_met_) results. Means and SEM (standard error of mean, grey area) of gross metabolic power over time (in minutes) have been plotted for the overground and the two treadmill trials. *OG* represents overground, *HTM* high treadmill and *LTM* low treadmill

Figure 2 provides an overview of means and SEM (grey area) of gross metabolic power for the elderly subjects for all the three walking conditions of overground, high treadmill, and low treadmill. Just as a first indication, for the difference between the means in the gross metabolic power to be statistically significant between the three walking conditions, it should be bigger than about twice the pooled SEM. Before doing any further statistical analysis, this figure shows clear differences between overground walking and the two treadmills, but not between the two treadmill conditions.

**Fig. 3:**
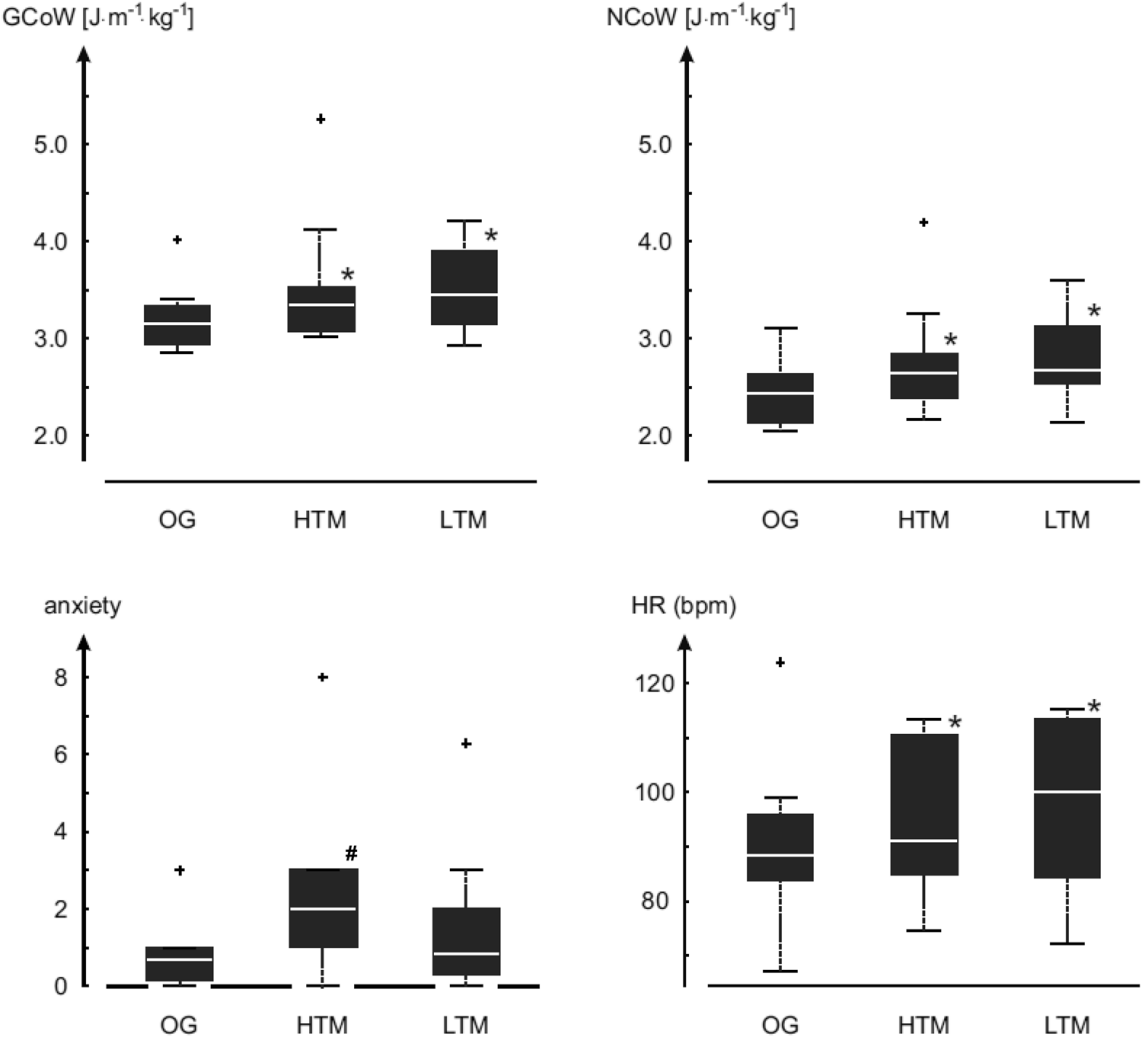
Boxplots of Gross Cost of Walking (GCoW), Net Cost of Walking (NCoW), self-reported anxiety, and Heart Rates (HR) are shown for overground (OG), high treadmill (HTM), and low treadmill (LTM) walking conditions. The central white horizontal lines in the boxplots represent the median and the black edges of the boxes represent the 25th and the 75th percentiles. The black whiskers of the boxplots extend up to the values that are not considered as outliers and the outliers are depicted by the black ‘+’ symbols. The ‘*’ symbols signify statistically significant elevation (*p* < 0.05) compared to overground values. For the self-reported anxiety plot, the ‘#’ symbol signify statistically significant elevation (*p* < 0.05) compared to overground and LTM values

Figure 3 provides the boxplot showing the median values and the interquartile range, along with the outliers and statistically significant results for the three walking conditions of overground, high treadmill, and low treadmill for MCoW, heart rates, and self-reported anxiety levels. These results are further discussed in detail in the following section.

### Statistical analyses

We found statistically significant differences in GCoW (*p* = 0.02) and NCoW (*p* = 0.002) between the three walking conditions (overground, high treadmill, and low treadmill). Post hoc analyses showed statistically significantly increased GCoW for high treadmill walking (6.03%, *p* = 0.03) compared to overground and for low treadmill walking (9.5%, *p* = 0.004) compared to overground. For NCoW, we again found a statistically significant increase for high treadmill walking (8.2%, *p* < 0.001) compared to overground and also for low treadmill walking (9.8%, *p* < 0.001) compared to overground. There were no statistically significant differences (*p* > 0.05) between the two treadmills for either GCoW or NCoW. This means that for elderly, there is an increased MCoW on treadmills compared to overground, irrespective of whether the treadmill is at a height or not.

In addition to these differences, statistically significant differences were found in self-reported anxiety levels (*p* = 0.02) between the three walking conditions. Post hoc analysis showed statistically significantly elevated anxiety for high treadmill walking (*p* = 0.004) compared to overground and for high treadmill walking compared to low treadmill walking (*p* = 0.02). There were no statistically significant differences (*p* > 0.05) in self-reported anxiety level between overground and low treadmill walking. In absolute numbers, self-reported anxiety had a median value of 2.0 on the high treadmill, 0.85 on the low treadmill, and 0.7 overground.

We also found statistically significant differences in heart rates (*p* = 0.03) between the three walking conditions. Post hoc analyses showed statistically significantly increased heart rates for high treadmill walking (2.7%, *p* = 0.009) compared to overground and for low treadmill walking (13.5%, *p* = 0.02) compared to overground. No statistically significant differences were found for heart rate values (*p* > 0.05) between high treadmill and low treadmill walking. These findings go against the subjectively measured higher anxiety on the high treadmill than the low treadmill, as stated earlier. To summarise, we see that there is a higher MCoW on the low treadmill (like the high treadmill), but no higher self-reported anxiety level compared to overground. Also, we see that higher anxiety on the high treadmill is not accompanied by higher MCoW, compared to low treadmill.

## Discussion

In this study, we recruited healthy, physically fit and active elderly, measured their PWS overground, and then imposed that speed on two treadmills—namely high treadmill placed at a height of 60 cm from the ground and low treadmill placed at 15 cm from the ground. We found an increased MCoW on both the treadmills compared to overground, thereby replicating our previous study (Das Gupta et al. 2021). Additionally, we found elevated self-reported anxiety on the high treadmill compared to overground, and on the high treadmill compared to the low treadmill, but not on the low treadmill compared to overground. This indicates that self-reported anxiety does not explain the increased MCoW on a treadmill for elderly observed in our current and previous study (Das Gupta et al. 2021). Differences in heart rate followed the results of MCoW: heart rate was increased on both treadmills and no significant differences were observed between the two treadmills. Below, we will discuss our results in detail and argue that it must be something other than self-reported anxiety that triggers the supposed gait changes leading to increased MCoW for elderly on a treadmill.

In the present study, we not only replicated our previous finding of a higher MCoW for elderly on high treadmill compared to overground (Das Gupta et al. 2021), but also showed that elderly have an increased MCoW on a low treadmill compared to overground. If we look at the literature, there are suggestions that anxiety could be a trigger for elderly to walk differently on a treadmill than overground (Parvataneni et al. 2009; Martin and Li 2017). This anxiety could be caused by unnatural visual flow on a treadmill (Dal et al. 2010; Martin and Li 2017; Murray et al. 1985) as there is a discrepancy between visual, vestibular, and other proprioceptive information. Increased anxiety can then lead to gait changes resulting in, for example, excessive muscle co-contraction which leads to an increased MCoW. In our previous study, we used a high treadmill that was elevated by 60 cm and this height could also (further) induce anxiety. We indeed observed that self-reported anxiety was higher for the high treadmill condition compared to the low treadmill. We did not observe elevation in self-reported anxiety in low treadmill walking compared to overground. Yet, MCoW did not differ between high treadmill and low treadmill, and were both elevated compared to overground. As such, we conclude that self-reported anxiety is not the trigger for gait changes that are responsible for the increased MCoW on a treadmill for elderly, but that apparently the treadmill itself is the trigger.

In this study, to be able to directly replicate our previous findings (Das Gupta et al. 2021), we did not randomize our conditions: all our participants first walked overground, then on the high, and then on the low treadmill. Therefore, it is possible that fatigue and familiarization influenced our results on MCoW and self-reported anxiety. To prevent fatigue, we provided at least 20 min rest before the start of a walking trial. Furthermore, it is likely that a (difference in) fatigue would have shown up in our RMR measurement directly prior to each condition, but no such difference was observed. In addition, none of the participants reported any physical fatigue after completion of a walking trial. Finally, if physical fatigue would have played a role, it would have been most pronounced in the last condition (low treadmill), which we did not observe. All in all, we are confident that the order of our conditions did not influence our MCoW results. It may well be, however, that familiarization during high treadmill walking caused a lower self-reported anxiety scores on the low treadmill and may be part of the reason of the differences in self-reported anxiety found between these two conditions. Be that as it may, we did not find a difference in MCoW between these two conditions, and thus, we feel it is safe to conclude that this difference in MCoW is not related to self-reported anxiety.

In the literature, it has been suggested that that the increased MCoW on a treadmill is due to anxiety, and this suggestion was based on observed changes in heart rate (Parvataneni et al. 2009). Here, we found that heart rates during walking on the two treadmills are comparable and that both are significantly higher than during overground walking. This is in line with our findings on MCoW: we observed no differences in MCoW between high treadmill and low treadmill, and we observed that MCoW was significantly higher on both treadmills than overground. Differences in heart rate were not in line with self-reported anxiety: we observed an increase in self-reported anxiety on high treadmill compared to low treadmill and on high treadmill compared to overground but, importantly, not on low treadmill compared to overground. Therefore, from these results, we conclude that heart rates reflect metabolic rate and not anxiety.

This study shows that the increase in NCoW for elderly on the high treadmill that we found in our previous study (Das Gupta et al. 2021) was not related to self-reported anxiety. At this point, we do not know what triggered the gait changes in treadmill walking in elderly that caused elevated MCoW. Nevertheless, here, we do show that MCoW measured during treadmill walking cannot be readily generalized to and directly compared with overground values for elderly, even when walking at the same walking speed. Since overground walking is ecologically more valid than treadmill walking, it follows that while studying human walking energetics, especially in elderly, the usage of a treadmill can be considered as a methodological confounder, as suggested in our previous study (Das Gupta et al. 2021). In our previous study (Das Gupta et al. 2021), we showed that for young adults, there were no differences between treadmill and overground MCoW. Due to this differential reaction of young and elderly to a treadmill, we recommend that for future research, effects of age on MCoW should not be studied on treadmills.

## Data Availability

The datasets generated during and/or analyzed during the current study are available from the corresponding author on reasonable request.

## Abbreviations

*d*: Cohen’s *d*
GCoW: Gross cost of walking
MCoW: Metabolic cost of walking
NCoW: Net cost of walking
PWS: Preferred walking speed
RER: Respiratory exchange ratio
RM-ANOVA: Repeated measures-analysis of variance
SD: Standard deviation
SEM: Standard error of mean
${\dot{\text{V}}\text{O}}_{2}$: Oxygen consumption rate
${\dot{\text{V}}\text{CO}}_{2}$: Carbon dioxide production rate
VAS: Visual Analogue Scale
